# Addressing Missing Permanent Premolars With Retained Deciduous Teeth: Orthodontic Solutions and Outcomes

**DOI:** 10.7759/cureus.63660

**Published:** 2024-07-02

**Authors:** Aathira Surendran, Pallavi Daigavane, Ranjit Kamble, Lovely Bharti, Mrudula Shinde, Aditya V Pareek

**Affiliations:** 1 Department of Orthodontics and Dentofacial Orthopedics, Sharad Pawar Dental College and Hospital, Datta Meghe Institute of Higher Education and Research, Wardha, IND

**Keywords:** fixed orthodontic treatment, missing maxillary 2nd premolars, missing premolars, retained deciduous molars, tooth agenesis

## Abstract

This case report presents an orthodontic treatment approach involving retained deciduous teeth. The patient presented with a Class I malocclusion and buccal crossbite. Despite the presence of retained deciduous teeth, a non-extraction treatment plan was devised to address the malocclusion and achieve optimal dental alignment.

The treatment protocol included the use of fixed appliances and quad helix to facilitate the alignment of permanent dentition. The progress of treatment was closely monitored through regular follow-up appointments and adjustments to the treatment plan as necessary.

Upon completion of treatment, the patient achieved a harmonious occlusion, improved dental alignment, and a pleasing aesthetic outcome. This case report highlights the successful management of a challenging orthodontic case through a non-extraction approach with retained deciduous teeth, emphasizing the importance of individualized treatment planning and careful consideration of each patient's unique dental anatomy.

## Introduction

Tooth agenesis is the most frequently occurring abnormality in tooth development. It can present itself either as a nonsyndromic solitary type or as a component of a hereditary syndrome [1]. The severity of tooth agenesis is determined by the number of missing teeth, with hypodontia referring to the absence of less than six teeth (excluding third molars), oligodontia referring to the absence of more than six teeth (excluding third molars), and anodontia indicating the absence of all teeth [2].

Tooth agenesis is a common condition that results in the absence of certain teeth at birth. The incidence of tooth agenesis varies between 2.6 and 11.3% of the population, depending on the country. The most commonly affected teeth are premolars and lateral incisors, accounting for over 80% of cases. This condition is associated with several dental problems, including extended retention, premature loss, infra-occlusion, and dental caries in primary dentition. Tooth agenesis can also cause clinical conditions such as anomalies in tooth size or form, delayed eruption, or abnormal eruption in permanent dentition [2].

The congenital absence of maxillary second premolars is a commonly occurring dental agenesis, ranking second in frequency after missing third molars. The mandibular second premolars are the most prevalent type of dental agenesis, followed by the maxillary lateral incisors [2,3]. Patients diagnosed with hypodontia frequently present with various dental malformations, including peg-shaped lateral incisors, impactions, transpositions, and retained deciduous teeth. In situations such as the one described, it is advisable to employ an interdisciplinary approach that encompasses both orthodontic and restorative therapies. This is a widely accepted and recommended course of action by professionals in the field, which has proven to yield favorable results [4].

Patients who present with multiple missing teeth often express apprehension about further extraction of healthy deciduous teeth, particularly if there is no guarantee of long-term benefit. Most dental professionals would agree that preserving deciduous teeth in an adolescent patient with a favorable facial profile and no crowding is preferable until natural exfoliation occurs. However, when a maxillary primary second molar is retained, the mesiodistal dimensions of the permanent premolar are smaller than the deciduous molars. Depending on these factors, the tooth could either be left in situ with a functional occlusion that may be less than ideal, or it may require reshaping to the size of a permanent premolar [5].

The patient under consideration is an adolescent whose congenital absence of maxillary second premolars presents a challenging orthodontic case. This study effectively demonstrates that retaining deciduous teeth in such situations can serve as a feasible treatment option, with favorable long-term outcomes.

## Case presentation

A 14-year-old female patient reported to the Department of Orthodontics and Dentofacial Orthopedics, Sharad Pawar Dental College and Hospital, Wardha, India, with a concern about forwardly placed upper anterior teeth. Based on the patient's medical records, there are no pre-existing conditions or history of facial or dental injuries that would hinder the feasibility of administering dental care.

**Figure 1:**
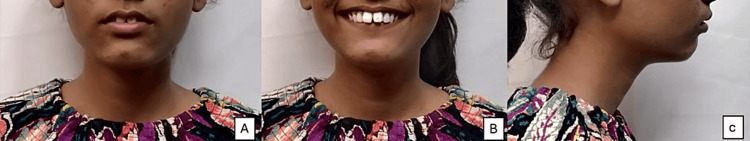
Pre-treatment extraoral photographs A) Frontal; B) Smiling; C) Profile

The extraoral features included a mesocephalic head form, a mesoprosopic facial form, and a bilaterally symmetrical face (Figure 1). Additionally, the profile is convex with posterior facial divergence, potentially competent lips, and an acute nasolabial angle. The clinical Frankfort-mandibular plane angle is average.

**Figure 2:**
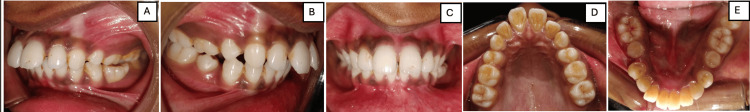
Pre-treatment intraoral photographs A) Left lateral; B) Right lateral; C) Frontal; D) Maxillary occlusal; E) Mandibular occlusal

Upon intra-oral examination, no abnormalities were discovered in relation to the alveolar ridge, tongue size and form, labial and buccal vestibule height, and gingival state. The permanent teeth are present, with an 8 mm overjet and a 4 mm overbite, as determined by the hard tissue examination. The molar relation on the right side is Class I, and the canine is in the end-of relation, while on the left side, it is Class II. A significant issue has been identified in the maxillary region involving the crossbite of a deciduous molar, with a narrowing of the maxillary arch. This issue requires our immediate attention, and we need to take appropriate measures to address it (Figure 2).

**Figure 3:**
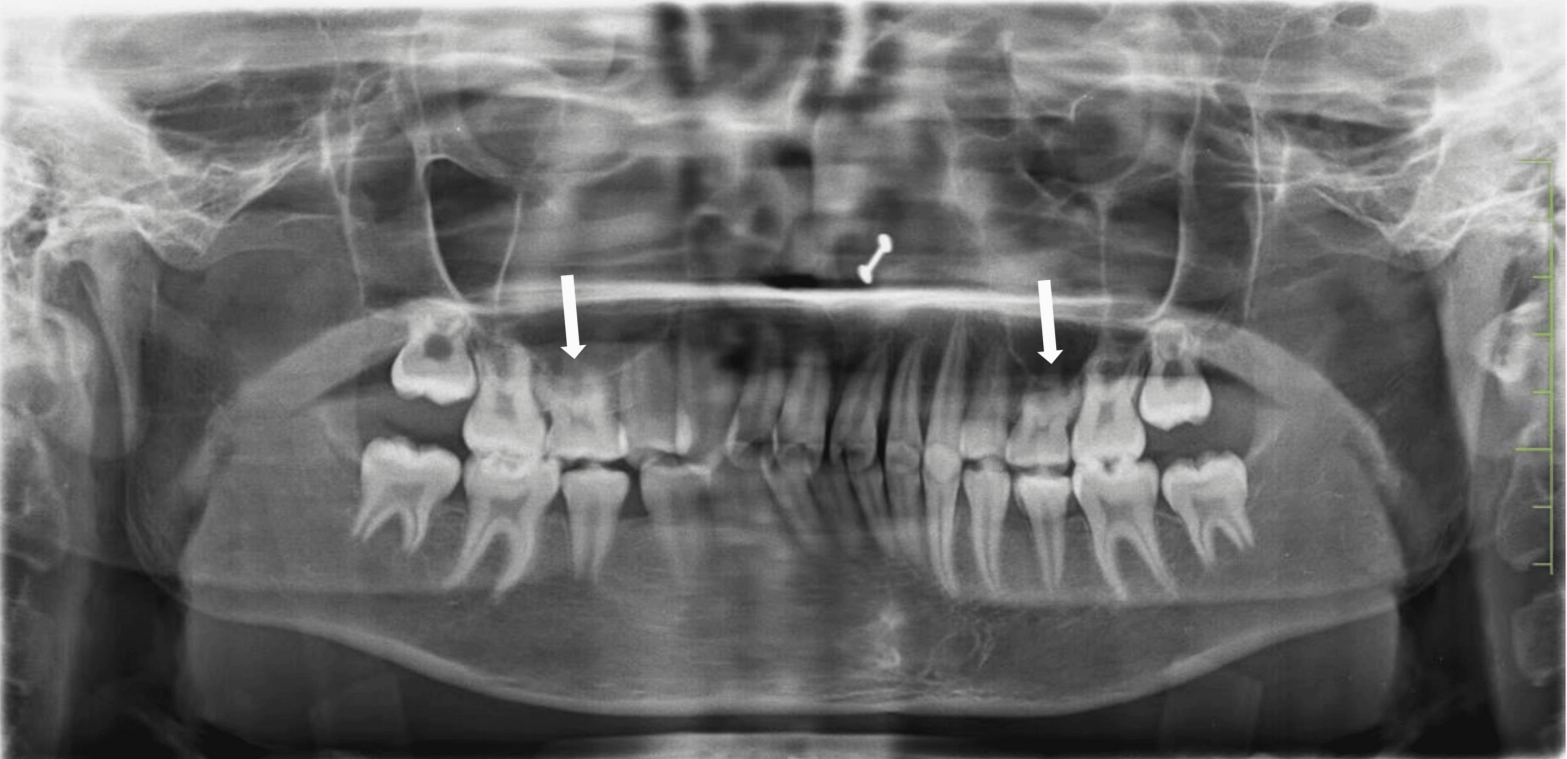
Pre-treatment orthopantomogram (OPG) The arrows show the presence of retained deciduous and the absence of premolar

**Figure 4:**
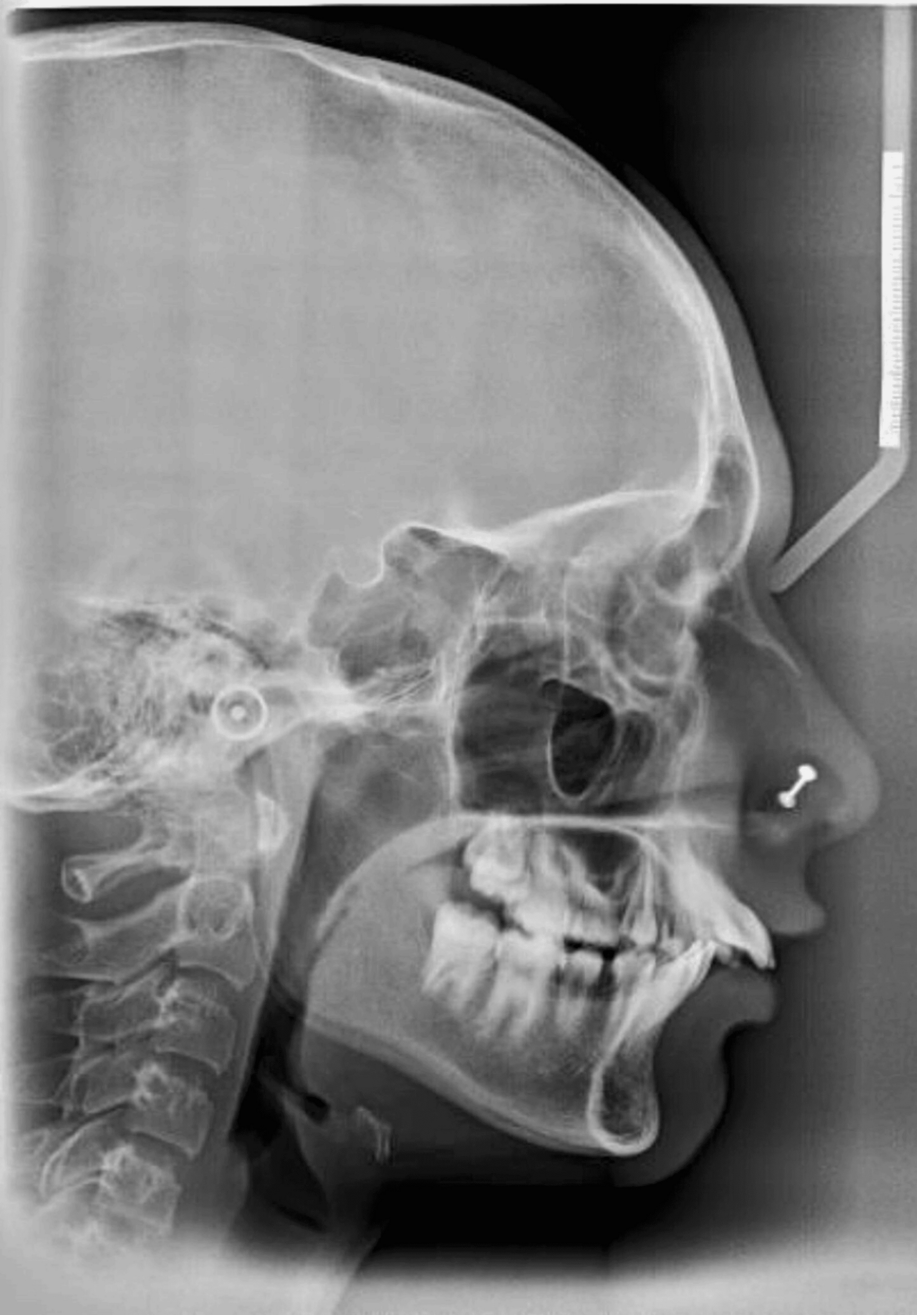
Pre-treatment lateral cephalogram

A pre-treatment orthopantomography (OPG) showed that teeth 15 and 25 (FDI - Federation Dentaire Internationale numbers) were congenitally missing, while teeth 55 and 65 were retained and not submerged in relation to their adjacent teeth (Figure 3). On the contrary, the mandibular arch exhibited the presence of all teeth. Moreover, the permanent second molars' tooth development in both the maxillary and mandibular arches was found to be closely matched. The patient's parents did not report any instances of missing teeth or related conditions in their family history. Based on the cephalometric evaluations, it was found that the patient had a normodivergent facial profile and a skeletal Class II relationship. The patient's facial profile was slightly convex, and no asymmetries were observed (Figure 4). The aforementioned diagnosis has been duly noted and will be taken into account when determining the most appropriate treatment protocol for the patient.

Treatment alternatives

The two possible treatment options to address a dental issue are as follows: the first option entails retaining the 65 and 55 deciduous teeth in their original positions due to their vertical alignment, immovability, and clinical stability. This course of action will lead to minimal resorption of the deciduous molar roots, while a quad helix will be utilized to expand the maxilla and correct the molar crossbite, thus realizing a stable and functional occlusion.

The second alternative involved slicing teeth 55 and 65 to that of a permanent second premolar. This approach is suitable for eventual implantation surgery and helps to maintain the buccolingual bone. However, the divergence of the roots limits the degree of remodeling possible with the deciduous teeth.

During the consultation, the patient was advised that the clinical stability of the remaining deciduous teeth did not necessarily imply a favorable long-term prognosis. To honor the patient's preference for minimal prosthetic therapy after orthodontic treatment, the final treatment plan incorporated the maxillary deciduous second molars, teeth 55 and 65. Following post-orthodontic treatment, periodic examinations of the remaining deciduous teeth were scheduled to ensure their continued stability.

Treatment progress

The case started with a pre-adjusted edgewise appliance (MBT 0.022" slot). The upper arch was bonded in the first month of therapy, except for the upper right and left second deciduous teeth, which remained unbonded throughout. The constriction of the arch that caused posterior crossbite was corrected by fabricating a quad helix and soldering it to the banded molars. The quad helix was employed to rectify the posterior crossbite and was activated for two months, and it was continued for an additional six months for retention.

**Figure 5:**
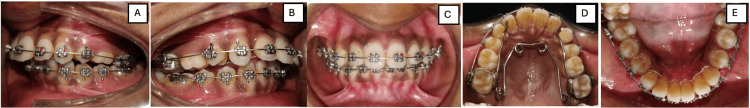
Intraoral photographs with quad helix A) Right lateral view; B) Left lateral view; C) Frontal; D) Maxillary occlusal view; E) Mandibular occlusal view

After a month of initial treatment, strapping of the lower arch was done. Alignment was started with 0.016" nickel-titanium wire in both the maxillary and mandibular arch. Subsequently, a thorough alignment and leveling process was carried out for a duration of three months, resulting in the successful correction of the crossbite (Figure 5). Initial leveling and alignment of the arches were started with round wires, followed by rectangular wire until 0.019 × 0.025 stainless steel wire. In order to correct the Class II canine and molar relation, Class II elastics were used: initially, pink elastics (size of 3/8" and 3.5 oz. force) for two months, followed by blue elastics (size of 1/4" and 4 oz. force) for four months. The arches were completely leveled and aligned without the need for extractions. After an 18-month course of treatment, the appliances were removed, and removable types of retainers were delivered.

Treatment results

**Figure 6:**
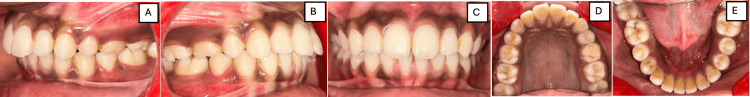
Post-treatment intra-oral images A) Left molar in occlusion; B) Right molar in occlusion; C) Frontal; D) Maxillary occlusal photograph; E) Mandibular occlusal photograph

**Figure 7:**
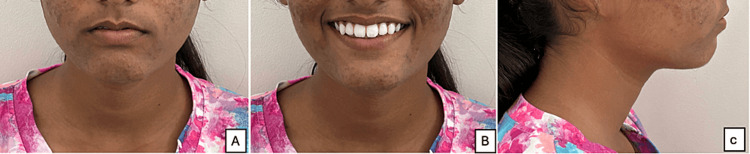
Post-treatment extra-oral images A) Frontal; B) Frontal smiling; C) Profile

After an 18-month therapeutic intervention, the treatment culminated in a Class I molar connection, while the mandibular premolars and the deciduous maxillary second molars exhibited good functional occlusion (Figure 6). The post-treatment extra-oral and intra-oral photographs revealed a marked improvement in profile, overjet of 2 mm, overbite of 2 mm, and crossbite correction. The therapy significantly enhanced the appearance of the smile, corrected the proclination of the upper and lower teeth, and fostered functional occlusion (Figure 7).

## Discussion

Hypodontia is a condition characterized by the absence of one or a few teeth. The mandibular second premolar is the most commonly missing tooth, aside from the third molars. Various treatment options exist for addressing permanent tooth hypodontia and retained deciduous teeth. It is important to weigh the advantages and disadvantages of extracting or preserving the deciduous tooth. Retention of the deciduous second molars is feasible if their long-term viability is expected, and if there is no significant crowding in the dental arch or protrusion of the facial soft tissues [2].

It has been observed that deciduous second molars with intact crowns, roots, and supporting alveolar bone that remain into adulthood can continue to function effectively for many years. This is promising for the long-term outlook of retained deciduous teeth [6]. Previous studies have demonstrated that retained deciduous molars, with premolar agenesis, have an excellent prognosis beyond the age of 20. However, evaluating the prognosis of retained deciduous teeth requires careful consideration of key factors such as infra-occlusion and root resorption [7]. Hvaring et al.'s study highlights the importance of infra-occlusion and root resorption in determining the prognosis of retained deciduous teeth. In their research, the deciduous maxillary second molars showed minimal root resorption and no signs of infra-occlusion [8].

The quad helix is used in orthodontics to expand the dental arch. It comprises a helix with four arms connected to bands that are cemented on the molars and bicuspids. By exerting pressure, this device gradually expands the arch, addressing crowding issues and enhancing tooth alignment. Primarily used in children and teenagers whose palates are still growing, the quad helix can also be used in adults. However, its effectiveness in expanding the palate may be somewhat limited in adults due to palatal sutures [9].

It was concluded that maintaining the primary teeth for as long as possible was the most prudent approach due to the patient's reluctance to undergo additional extractions. This strategy can help prevent atrophic changes in the alveolar bone and periodontal ligament, as long as the primary teeth maintain proper occlusal function. Furthermore, the even distribution of occlusal forces can minimize excessive stress on the retained primary teeth, promoting long-term stability. The patient has notably responded well to this treatment strategy, focused on preserving the primary teeth.

## Conclusions

Patients with retained deciduous teeth may face challenges such as tooth agenesis, impactions, transpositions, and deformities, complicating the development of a final treatment plan, particularly in growing children. However, early diagnosis, prompt intervention, and a multidisciplinary approach that combines restorative and orthodontic care can significantly improve the quality of care and expand treatment options. This case demonstrates that it is possible to integrate retained deciduous teeth with a favorable prognosis into the final occlusion, resulting in positive long-term clinical outcomes.
